# Physiological Observations on the Pulsations of the Heart, and Its Diurnal Revolutions and Excitability

**Published:** 1837

**Authors:** 


### II.—ANALYTICAL.

---

Art. I.—Physiological Observations on the Pulsations of the Heart, and its Diurnal Revolutions and Excitability. By Dr. Knox, of Edinburgh.

In Bell's Journal, for August last, we have a paper on the above-named subject, by Dr. Knox, read before the Royal Society of Edinburgh. Being too long to reprint under our Analectic head, we shall extract the conclusions to which the author arrives, which are the following:

*General Conclusions.*—It is certainly much safer for the reader to draw from the foregoing tables and observations whatever conclusions he may think fit, or that he thinks the data will bear out; but least it be said that I have come to no conclusion, (which those are apt to say who have no patience to think for themselves,) I shall venture a few, begging it, however, to be distinctly understood that the observations may themselves warrant quite different ones.

1. The velocity of the heart's action is in the direct ratio of the age of the individual, being quickest in young persons, slowest in the aged. There may be exceptions to this, but they do not affect the general law.

2. There are no data to determine the question of an average pulse for all ages.

3. There is a morning acceleration and an evening retardation in the number of the pulsation of the heart, independent of any stimulation by food, &c.

4. The excitability of the heart undergoes a daily revolution; that is, food and exercise most affect the heart's action in the morning and during the forenoon, least in the afternoon, and least of all in the evening. Hence we should infer that the pernicious use of spirituous liquors must be greatly aggravated in those who drink before dinner.

5. Sleep does not farther affect the heart's action than by a cessation of all voluntary motion, and by a recumbent position.

6. In weak persons, muscular action excites the action of the heart more powerfully than in strong and healthy individuals; but this does not apply to other stimulants, to wine, for example, or to spiritous liquors.

7. The effects of the position of the body in increasing or diminishing the number of pulsations is solely attributable to the muscular exertion required to maintain the body in the sitting or erect position; the debility may be measured by altering the position of the person from a recumbent to the sitting or to the erect position.

8. The law of the differential pulse is not universal. There are exceptions to be found even in those in perfect health. It is also possible that there may be some in whom the diurnal revolutions of the pulse takes place only in consequence of the use of stimulants. But this has not been proved satisfactorily.

9. The most powerful stimulant to the heart's stimulating action is muscular exertion. The febrile pulse never equals this.

10. The law of relation between inspiration and the pulsation of the heart has been stated by M. Quetelet.

Most of these conclusions are in coincidence with those to which the profession had already come. In regard to the 3d, it may be remarked, that it refers merely to the frequency of the pulse. Its *momentum* is, undoubtedly, greater in the evening than the morning; and hence patients bear the loss of blood much better in the former than the latter period. D.

